# Methamphetamine Use in Manitoba: An Evidence-to-Action (E2A) approach to a linked administrative data study.

**DOI:** 10.23889/ijpds.v7i3.1933

**Published:** 2022-08-25

**Authors:** Amy Freier, Carolyn Shimmin, Mariette Chartier, Jennifer Enns, Scott McCulloch, The Methamphetamine Use In Manitoba Research Team, Nathan Nickel

**Affiliations:** 1 Manitoba Centre for Health Policy; 2 Centre for Healthcare Innovcation

## Objectives

A multi-disciplinary E2A group was established as part of a linked administrative data study examining methamphetamine use in Manitoba. In this presentation we will share our experiences of establishing an E2A, embedding stakeholders in the research process, and outline an iterative engagement plan for the remaining study years.

## Approach

The E2A group is led by two researchers with expertise in patient and public engagement and guided by Pal’s (2014) work on policy analysis and activation. Pal emphasizes a multidisciplinary and iterative process as the basis of a more inclusive approach to policy development for complex problems, such as the prevalence of methamphetamine use in Manitoba. Our goal in engaging public rightsholders, service providers and knowledge users in the research is to ensure that their first-hand knowledge and perspectives are reflected in the interpretations of the findings and that analyses address identified complexities in a culturally sensitive and equity-focused way.

## Results

In the fall of 2020 E2A members were recruited, including persons with lived experience using methamphetamines and their families/care-givers, academics, clinicians, government, and non-government stakeholders. Training was provided on the topics of trauma-informed care, public engagement, effects of colonial and racist institutions, and cultural safety. The first E2A meetings co-developed guiding principles, a vision and mission, as well as provided capacity building around administrative data research. This work was done as a foundation for the next two years, wherein the group will make decisions about key variables (ie: between methamphetamine use and mental health), interpretation of results, knowledge mobilization, and policy development using an iterative self-evaluation process. To date challenges addressed included public health restrictions related to Covid-19 and adapting the research flow to centre lived-experience decision making.

## Conclusion

The E2A group is a key component of this study and could serve as a model for other administrative data studies. The group prioritizes stakeholder knowledge, interprets results, flags potential biases in the data research process, and ensures the findings are relevant to the people they are meant to support.

